# Case report: Comprehensive follow-up of a Colombian family carrying a novel MEN1 variant linked to a rare ACTH-producing pancreatic neuroendocrine carcinoma

**DOI:** 10.3389/fendo.2024.1398436

**Published:** 2024-07-22

**Authors:** Julián C. Riaño-Moreno, Angélica María González-Clavijo, William C. Torres J., Vilma L. Medina B., Alfredo Ernesto Romero-Rojas, Isabella Vieda-Celemin, Jordan A. Avila-Moya, Johan A. Baron-Cardona, Juan P. Bravo-Patiño, Oscar S. Torres-Zambrano, Luis Felipe Fierro Maya

**Affiliations:** ^1^ Department of Pathology and Molecular Oncology, Instituto Nacional de Cancerología, Bogotá, Colombia; ^2^ Faculty of Medicine, Universidad Cooperativa de Colombia, Villavicencio, Colombia; ^3^ Department of Bioethics, Universidad El Bosque, Bogotá, Colombia; ^4^ Department of Physiological Sciences, Faculty of Medicine, Universidad Nacional de Colombia, Bogotá, Colombia; ^5^ Endocrine Oncology Unit, Instituto Nacional de Cancerología, Bogotá, Colombia

**Keywords:** multiple endocrine neoplasia type 1 (MEN1), cascade genetic screening, neuroendocrine tumors (NETs), frameshift variant, neuroendocrine - carcinoma

## Abstract

**Background:**

Multiple Endocrine Neoplasia type 1 (MEN1) is an autosomal dominant disorder marked by pathogenic variants in the MEN1 tumor suppressor gene, leading to tumors in the parathyroid glands, pancreas, and pituitary. The occurrence of ACTH-producing pancreatic neuroendocrine carcinoma is exceedingly rare in MEN1.

**Case presentation:**

This report details a Colombian family harboring a novel MEN1 variant identified through genetic screening initiated by the index case. Affected family members exhibited primary hyperparathyroidism (PHPT) symptoms from their 20s to 50s. Uniquely, the index case developed an ACTH-secreting pancreatic neuroendocrine carcinoma, a rarity in MEN1 syndromes. Proactive screening enabled the early detection of pituitary neuroendocrine tumors (PitNETs) as microadenomas in two carriers, with subsequent surgical or pharmacological intervention based on the clinical presentation.

**Conclusion:**

Our findings underscore the significance of cascade screening in facilitating the early diagnosis and individualized treatment of MEN1, contributing to better patient outcomes. Additionally, this study brings to light a novel presentation of ACTH-producing pancreatic neuroendocrine carcinoma within the MEN1 spectrum, expanding our understanding of the disease’s manifestations.

## Introduction

Multiple Endocrine Neoplasia type 1 (MEN1) is an autosomal dominant disorder with a global prevalence ranging from 1 to 10 cases per 100,000 individuals, showing no preference for gender. Its penetrance increases significantly with age, being reported at 50% by age 20, exceeding 95% by age 40, and nearing 100% beyond age 70 (1). The condition primarily results from loss-of-function variants in the MEN1 gene on chromosome 11q13. This gene encodes Menin, a 610-amino acid scaffold protein that plays a crucial role in various cellular processes. Menin interacts with several intracellular molecules, such as JunD, NFκB, and Smad3, implicating it in transcriptional regulation, maintaining genome stability, and controlling cell division and proliferation (2).

MEN1 is characterized by the development of primary hyperparathyroidism (PHPT) in 95% of cases, pituitary neuroendocrine neoplasms (PitNENs) in 30–40%, and duodenal-pancreatic neuroendocrine tumors (DP-NETs) in 40–70% of patients (3). Additionally, the presence of carcinoids, adrenocortical tumors, and facial angiofibromas highlight the syndrome’s clinical variability.

The diagnosis of MEN1 is complex, dependent on either the identification of two or more MEN1-associated tumors, finding a MEN1 tumor in a patient with a first-degree relative who has a MEN1 pathogenic variant, or uncovering an asymptomatic carrier via genetic cascade screening. Given MEN1 protracted natural progression, early-stage diagnosis presents significant challenges. This difficulty is compounded by clinical variability and the interplay of genetic and environmental factors, highlighting the crucial role of genetic testing. Such testing is instrumental in not only confirming MEN1 diagnoses but also in identifying individuals at risk, ensuring timely intervention and management (4).

Here, we present a comprehensive 14-year follow-up of a Colombian family affected by a novel pathogenic variant MEN1: c.698dup, p.Met233IlefsTer4. This genetic variant impacted five family members across three generations, discovered through cascade genetic screening (CGS) initiated after identifying the index case. Notably, the index case manifested with an adrenocorticotropic hormone (ACTH)-producing pancreatic neuroendocrine carcinoma, leading to ectopic Cushing’s syndrome—a presentation not previously reported in MEN1.

## Materials and methods

### Genetic test and analysis

Molecular analysis of the MEN1 gene was conducted at the Instituto Nacional de Cancerología, Bogotá D.C., Colombia, utilizing Sanger sequencing to analyze genomic DNA from peripheral lymphocytes of the index case and consenting asymptomatic family members. We targeted the entire coding sequence and exon-intron junctions of the MEN1 gene, amplifying nine exons with eight primer pairs. The amplified products were sequenced using the BigDye Terminator Cycle Sequencing Kit v3.1 on an Applied Biosystems/HITACHI 3500 Genetic Analyzer, USA, according to standard protocols.

Electropherograms were analyzed against reference sequences NG_008929.1 and NM_130799.2 using SeqA v6.0 and SeqScape v3.0 software from Applied Biosystems, USA, ensuring precise variant identification. Variant analysis followed the American College of Medical Genetics and Genomics (ACMG) guidelines (5).

Additionally, *in silico* analyses predicted the impact of frameshift indels using the SIFT Indel tool (6). The potential for nonsense-mediated decay (NMD) induced by the variant was assessed with the ‘masonmd’ R package by Hu, Yau, and Ahmed (7). For structural analysis and visualization, the PyMOL Molecular Graphics System by DeLano Scientific, San Carlos, CA, USA, was used.

### Index and carries clinical follow-up

Our protocol for monitoring asymptomatic MEN1 variant carriers adheres to the Endocrine Society’s guidelines (8), incorporating biochemical and radiological tests specified for different tumor types from certain ages, as outlined in **Tables 1** and **2**. For parathyroid tumors, annual screenings of calcium and PTH start at age eight, bypassing imaging. DP-NET screenings—gastrinomas from age 20 with gastrin and gastric pH tests, insulinomas from age 5 with fasting glucose and insulin levels, and other NETs from age 10 with Chromogranin-A and additional markers—are supported by yearly imaging (MRI, CT, or EUS). Pituitary tumors are screened from age 5 using prolactin and IGF-I levels, plus triennial MRI scans. Adrenal screenings, initiating at age 10, involve yearly MRI or CT scans, triggered by specific symptoms or when detecting tumors exceeding 1 cm.

**Table 1:** Clinical and biochemical findings in family members with MEN1 during the follow-up.

	III-2	II-4	III-3	III-4	IV-1
*MEN1* Sanger sequencing	+	+	+	+	+
Age at diagnosis (years)	24	48	21	23	1
PHPT	+	+	+	+	ND
Age at diagnosis (years)	24	55	23	24	
PTH (pg/ml) (reference value)	93.29 ↑ (15-65)	156 ↑↑ (15-65)	122 ↑↑ (15-65)	156 ↑↑ (15-65)	44 (15-65)
Calcium (mg/dl) (reference value)	11.1 ↑ (8.4-10.2)	13 ↑ (8.4-10.2)	10.8 ↑ (8.4-10.2)	11.2 ↑ (8.4-10.2)	9.6 (8.4-10.2)
PitNEN	ND	ND	+	+	ND
Pituitary MRI	Normal	Normal	Pituitary Microadenoma	Pituitary Microadenoma	Normal
Age at diagnosis (years)			23	25	
Prolactin (ng/ml) (reference value)	26.47 (1.3-25)	23.3 (4.7-23.3)	153.6 ↑↑↑ (1.3-25)	133 ↑↑↑ (1.3-25)	17.2 (1.3-25)
IGF-1 (ng/ml) (reference value)	–	298 (135-449)	232.5 (116-358)	581 ↑↑↑ (116-358)	292 (286-660)
OGTT-induced GH nadir (ng/ml) (reference value)				1.51 ↑↑↑ (<0.4)	
DP-NETs	+	+	ND	ND	ND
Age at diagnosis (years)	24	56			
Abdominal image findings	Pancreas tumor Liver metastases	Duodenal tumors Nodal metastases	Normal	Normal	Normal
Functional DP tumors	Hyperinsulinemic hypoglycemia (insulinoma) and ectopic Cushing´s syndrome*	Zollinger-Ellison syndrome (gastrinoma)			

(+), presence; (-): absence. DP-NETs, duodenal-pancreatic neuroendocrine tumors; GH, growth hormone; IGF-1, insulin-like growth factor 1; MRI, magnetic resonance imaging; ND, Non-Diagnostic; OGTT, oral glucose tolerance test; PHPT, primary hyperparathyroidism; PTH, parathyroid hormone; PitNEN, pituitary neuroendocrine neoplasms; Images used include MRI images for all subjects except III-3 and IV-1 for whom abdominal ultrasonography was used as an alternative. *Ectopic Cushing’s syndrome caused by a pancreatic neuroendocrine carcinoma metastatic to the liver. Empty cells indicate not to apply. Data for member IV-1 derived from the most recent follow-up at the age of 12 years.

**Table 2:** Biochemical findings in patients with functional DP-NETs.

	III-2	II-4
Insulinoma	+	-
Insulin (uU/ml) (Cut-off to diagnosis)	3.71 (>3)	–
Proinsulin (pmol/l) (Cut-off to diagnosis)	34.9 (>5)	–
C-peptide (ng/ml) (Cut-off to diagnosis)	1.12 (>0.6)	–
Glucose (mg/dl) (Cut-off to diagnosis)	27 (<55)	118 (<55)
Gastrinoma	-	+
Gastrin (pg/ml) (Cut-off to diagnosis)	–	> 1000 (>125)
Ectopic Cushing’s syndrome	+	-
ACTH (pg/ml) (Cut-off to diagnosis)	78.95 (>15)	–
Cortisol DST (ug/dl) (Cut-off to diagnosis)	120 (<1.8)	–

(+), presence; (-), absence. ACTH, adrenocorticotropic hormone; DST, dexamethasone suppression test.

## Patients and results

### Index case III-2

The index patient, III-2, a 24-year-old female, exhibited symptoms of hyperinsulinemic hypoglycemia for a year before being diagnosed in March 2009. Symptoms included asthenia, adynamia, syncopal episodes, and a seizure a month prior to diagnosis. An endoscopic ultrasound identified a focal hypoechoic lesion with regular edges, measuring 18 mm, located at the pancreas’s head and body junction.

In April 2009, a partial pancreatectomy and splenectomy were performed, revealing a multifocal, well-differentiated grade 1 NET according to the WHO 2000 criteria. Reclassification under the WHO 2022 criteria would correspond to a Grade 1 neuroendocrine tumor (well-differentiated, 0 mitoses per 2 mm², and a Ki67 proliferation index of 1%).

The immunohistochemistry (IHC) studies performed showed the tumor was positive for CKA1AE3, chromogranin, synaptophysin, and focal positivity for insulin. The mitotic rate was 0 per 2 mm², and the Ki67 index was 1%, indicative of an insulinoma. Examination confirmed no necrosis, vascular invasion, or extrapancreatic extension. The pancreatic section edge, spleen, and a peripancreatic lymph node were all tumor-free. Examination confirmed no necrosis, vascular invasion, or extrapancreatic extension. The pancreatic section edge, spleen, and a peripancreatic lymph node were all tumor-free.

After eight months free of hypoglycemic symptoms, the patient developed tonic-clonic seizures and hyperinsulinemic hypoglycemia again. New abdominal imaging showed another lesion in the pancreatic head. An octreotide scintigraphy (99mTc-OCTREOTIDE HYNIC) did not show any hypercaptating lesions expressing somatostatin receptors in the gastroenteropancreatic region or other organs (no image available).

This led to further surgery at the Instituto Nacional de Cancerología, Bogotá D.C., Colombia, including a residual pancreatectomy, duodenal resection, and peripancreatic soft tissue resection in May 2010. Pathology confirmed a well-differentiated, grade 1 NET (WHO 2010 criteria), with two lymph nodes involved, one positive for gastrin and ACTH (**Table 2**).

The subsequent development of PHPT was surgically addressed with subtotal parathyroidectomy and reimplantation. After this intervention, the patient required insulin therapy for the management of post-surgical diabetes mellitus.

**Figure 1:**
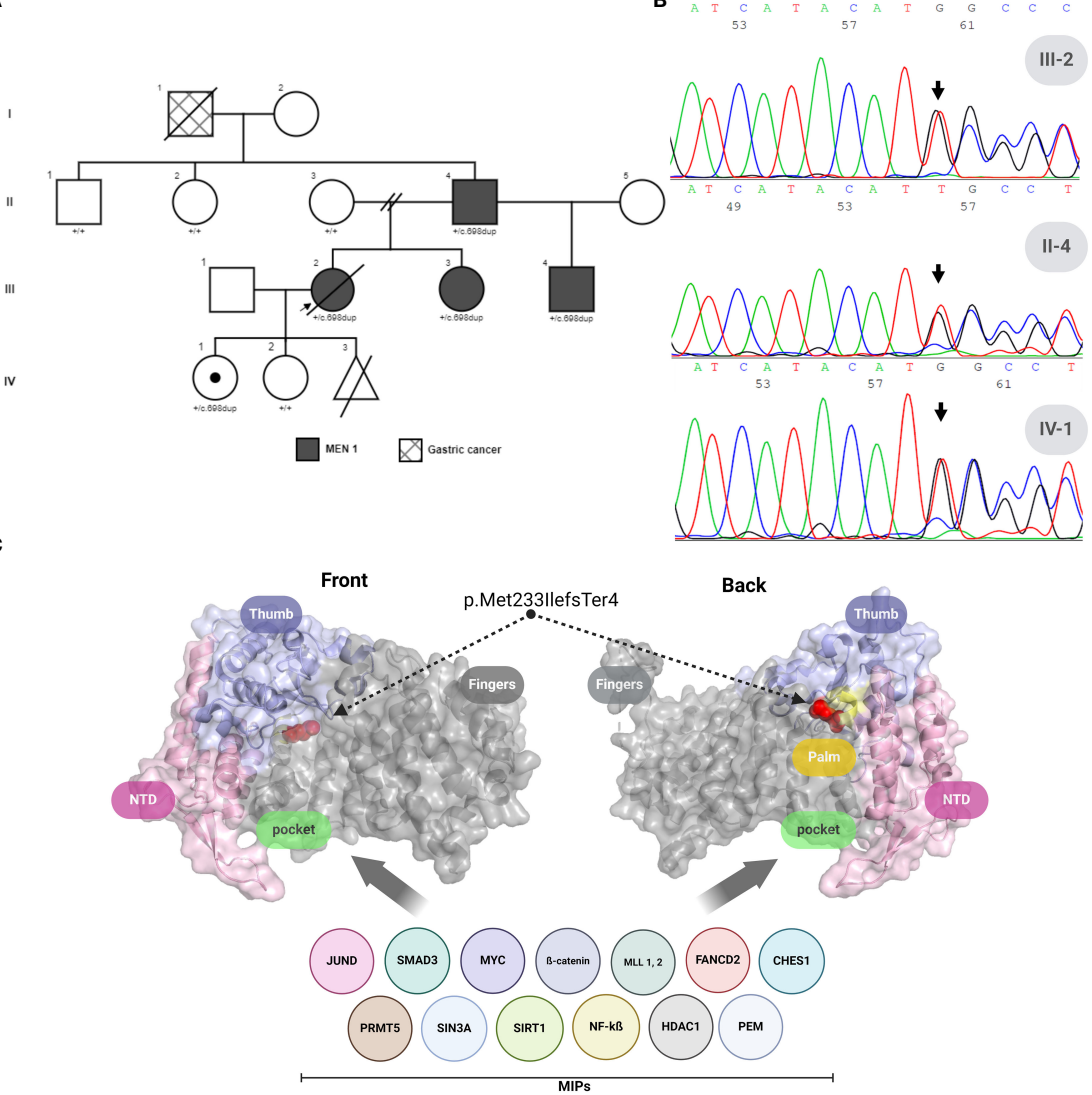
*MEN1* variant analysis and menin protein structure in a colombian family. **(A)** Family pedigree across four generations highlighting the index patient (arrow). **(B)** Forward Sanger sequencing results for the proband (III-2), the proband’s father (II-4), and the proband’s daughter (IV-1), indicating the presence of the MEN1: c.698dup frameshift variant. **(C)** Three-dimensional crystal structure of human Menin (PDB ID: 3U84), frontal and back views. Domains are color-coded: N-terminus (light pink), Thumb (light violet), Palm (pale yellow), with regions affected by MEN1: c.698dup, p.Met233IlefsTer4 highlighted in gray. The critical Met233-to-isoleucine mutation within the compromised domains is marked in red, affecting the Palm and complete Finger domain, and illustrating the disruption’s impact on MIPs interaction regions. MIPs, Menin interaction proteins. Created with BioRender.com.

The identification of multiple DP-NETs and parathyroid adenomas prompted the suspicion of MEN1, resulting in a referral for genetic testing in July 2010. Sanger sequencing identified a novel heterozygous variant in the MEN1 gene: c.698dup, p.Met233IlefsTer4. This finding initiated CGS within the family (**Figure 1A**).

The patient lost contact in 2011 due to pregnancy but returned in 2013 with hyperglycemia and severe hypokalemia, leading to a diagnosis of ACTH-dependent ectopic Cushing’s syndrome (EAS). Imaging (abdominal and pelvic MRI) showed focal hepatic lesions and pelvic bone involvement but no pancreatic or thoracic lesions. An octreotide scintigraphy (99mTc-OCTREOTIDE HYNIC) performed in June 2013 before the surgical intervention did not show any lesions expressing somatostatin receptors.

Treatment with ketoconazole partially controlled hypercortisolism, and a left hemihepatectomy with periportal lymph node resection was performed in June 2013 to address liver compromise. Pathology revealed liver and periportal lymph node metastases from a poorly differentiated small cell neuroendocrine carcinoma (WHO 2010 criteria). Reclassification under the WHO 2022 criteria would correspond to a small cell neuroendocrine carcinoma (25 mitoses per 2 mm² and a Ki67 index of 50%), with focal ACTH positivity in both the liver lesion and metastatic nodes.

Other causes of ACTH-producing tumors were excluded by performing comprehensive imaging studies, including brain MRI, chest CT, and abdominal-pelvic MRI. Additionally, an octreotide scintigraphy (HYNIC TOC) was conducted, revealing no other tumor lesions apart from those in the liver and a bone lesion in the pelvis. A PET 68-Ga-Dota scan was not performed due to unavailability, but a HYNIC TOC scintigraphy was done prior to the left hemi-hepatectomy, and the liver lesion did not express somatostatin receptors, confirming the cause of EAS as ACTH-producing tumors.

Despite external radiotherapy and chemotherapy with etoposide/cisplatin, the disease progressed to involve the liver, bones, and lungs, leading to the patient’s death five years post-diagnosis.

### Variant analysis and curation

The novel 698-base pair (T) duplication in the *MEN1* gene was identified via Sanger sequencing (**Figure 1B**), resulting in a significant frameshift variant designated as *MEN1* (NM_000244): c.698dup, p.Met233IlefsTer4 (**Figure 1B**). This frameshift variant affects transcript NM_000244.3 (NCBI) and an alternative transcript, ENST00000377316.6 (Ensembl). It remains unreported in ClinVar, the French MEN1 database (http://www.umd.be/MEN1/), and other genomic variation databases (1000Genomes, ExAC, dbSNP, and HGMD).

Protein modeling, as shown in **Figure 1C**, predicted a compromise in the Palm and Finger domain of the Menin protein (PDB ID: 3U84), which affects the Pocket region—a well-known interaction area with other significant oncogenic proteins. Given the variant’s class (insertion/duplication), we employed the SIFT Indel tool (6). Using sequence data based on GRCh37/hg19, we predicted that our variant is likely to be damaging, with a confidence score of 0.858. Additionally, this tool uses a basic criterion for predicting nonsense-mediated decay (NMD), suggesting that our variant could trigger NMD due to its location within the transcript.

Further analysis with the algorithm developed by Hu, Yau, and Ahmed (7), using ENTREZ ID: 4221, classified the variant as likely to elicit NMD. This classification takes into account criteria such as having more than two exons (our case 9 exons), the distance of the stop codon from the last exon-exon junction being greater than 50 bp (in our case, 1365 bp), and the premature termination codon (PTC) being located more than 200 bp downstream from the start codon (in our case, 856 bp), resulting in a mutated coding sequence length of 1850 nt. Our variant meets these criteria, further supporting its potential to initiate NMD.

Following the analysis, the variant was initially classified as “likely pathogenic” based on the phenotype of the index case, aligning with PVS1 and PM2 criteria. Subsequently, the detailed follow-up, which demonstrated the variant’s familial segregation (meeting the PP1 criterion) and the appearance of MEN1 symptoms in carriers (fulfilling the PP4 criterion), led to an upgrade in its classification to “pathogenic.” This revision highlights the significant correlation between the observed clinical features and a disease with a genetic foundation (9).

### Cascade genetic screening

**Figure 2:**
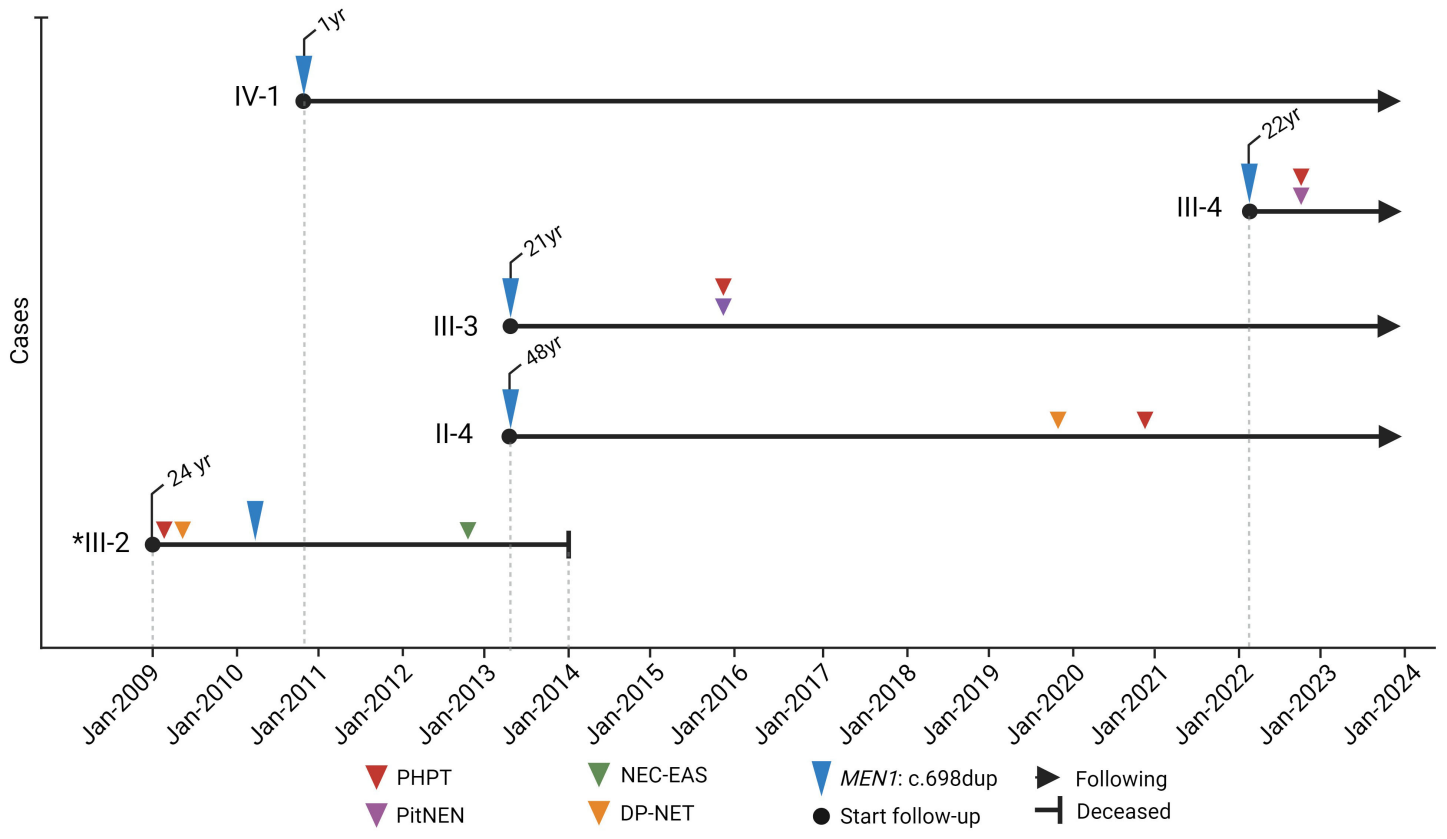
Timeline of molecular and clinical manifestations of MEN1 in the colombian family with MEN1: c.698dup. *index case. Created with BioRender.com.

From 2010 to 2022, a cascade genetic screening, preceded by pretest genetic counseling, was conducted on eight family members spanning three generations (**Figure 1A**), utilizing Sanger sequencing. This screening uncovered four asymptomatic carriers of the pathogenic variant *MEN1*: c.698dup, p.Met233IlefsTer4. The initial screening in 2010 focused on the daughters of the index case, leading to the identification of one carrier (IV-2). Further testing of her parents revealed that her mother was not a carrier, while her father (II-4) was found to be a carrier in 2013. In the same year, screenings were extended to the paternal aunts of the index case, all of whom tested negative. However, the fraternal sister of the index case was also identified as a carrier. After II-4’s remarriage and the birth of a son, the child, given the family’s medical history, was tested in 2021 and confirmed as a carrier of the MEN1 variant. Following the identification of this variant, clinical follow-up started after post-test genetic counseling was provided (**Figure 2**).

### Follow-up MEN1 carriers

#### Case II-4

Patient II-4, the father of the index case, was identified as a *MEN1* variant carrier on June 21, 2013 (**Figure 2**), at 48 years old but remained asymptomatic until 2020, at age 55, when he began experiencing dyspepsia and self-limited diarrhea. This led to the discovery of hypergastrinemia. An upper digestive tract endoscopy uncovered a nodular lesion in the duodenum, and a biopsy confirmed well-differentiated, multifocal, grade I NETs. Abdominal MRI later revealed a lesion adjacent to the uncinate process and another near the third portion of the duodenum, both without compressive effects, along with an intramural lesion in the second portion of the duodenum. The disease was considered unresectable not only due to the duodenal mass but also because of conglomerated lymph nodes around the pancreas and duodenum. Given the inoperability, treatment was initiated with proton pump inhibitors and Lanreotide, stabilizing the disease for a year. However, in January 2022, disease progression was observed, leading to the administration of four cycles of lutetium-177 DOTATOC treatment, totaling a dose of 800 mCi by September 2022, which resulted in stable disease six months post-treatment.

Additionally, in early 2021, PHPT was diagnosed, leading to a left lower parathyroidectomy in March 2021. Histopathological examination confirmed a parathyroid adenoma, achieving biochemical control post-surgery. As of the last follow-up, there has been no documented pituitary involvement.

#### Case III-3

Patient III-3, the sister of the index case, was identified as a *MEN1* variant carrier in June 2013 at 21 years old (**Figure 2**). Three years after her identification, she was diagnosed with a prolactin-secreting pituitary microadenoma, prompting the initiation of cabergoline treatment. Concurrently, PHPT was diagnosed, leading to the surgical intervention of right lower parathyroidectomy. These treatments successfully controlled her prolactin and calcium levels.

In June 2020, a follow-up pituitary MRI revealed no visible lesion, leading to the discontinuation of cabergoline. However, six months later, she experienced a recurrence of symptomatic hyperprolactinemia, necessitating the resumption of cabergoline therapy. Now, at 31 years old, she remains under treatment, and as of the latest assessments, no duodenal-pancreatic neuroendocrine tumors (NETs) have been detected.

#### Case III-4

Patient III-4 was confirmed as a *MEN1* variant carrier in March 2022 at 22 (**Figure 2**). Following the discovery, the patient consulted with endocrinology and genetic services. Eight months after variant identification, screening unveiled a lobulated pituitary adenoma measuring 9 x 9 x 9 mm. Biochemical evaluations indicated excesses in growth hormone (GH) and prolactin. Consequently, a transsphenoidal surgical approach was employed to remove the pituitary lesion within the same year. Histology of the excised tissue revealed a pituitary microadenoma with a focal expression of prolactin and growth hormone and a Ki67 proliferation index of 3%. Following surgery, the patient developed central hypocortisolism, necessitating the initiation of hydrocortisone supplementation. Despite surgery, persistently elevated insulin-like growth factor 1 (IGF-1) and GH levels were observed, leading to the prescription of somatostatin analog treatment.

In parallel, PHPT was diagnosed with scintigraphy detecting a left lower parathyroid lesion. The patient is currently awaiting surgical intervention for this condition. As of the last evaluation, no duodenal-pancreatic NETs have been identified.

#### Case IV-1

Patient IV-1 (**Figure 2**), the daughter of the index case, was confirmed as a *MEN1* variant carrier in December 2010 when she was only one year old. Now, at the age of 14, she continues to be asymptomatic, with no tumors detected through ongoing monitoring.

## Discussion

Here, we present clinical monitoring of a Colombian family where a novel frameshift variant in the MEN1 gene is present across three generations. The variant, MEN1: c.698dup, p.Met233IlefsTer4, was classified as pathogenic using ACMG criteria and in silico tests and observed clinical features within the family.

Although frameshift variants constitute the most prevalent alterations in MEN1, accounting for 42% of cases (10), the precise molecular mechanisms underlying their impact remain varied and undefined. Welch et al. (11) described a frameshift variant, c.674delG, located in exon 4 of MEN1, merely 8 codons proximal to our identified variant. This variant was identified in four family members diagnosed with PHPT and DP-NETs, with one member also presenting additional tumor types. Despite certain clinical resemblances with our case, the manifestations in our family exhibit greater variability, which might be attributed to variances in clinical follow-up protocols and the specific molecular mechanisms of the variant.

Welch et al. (11) attributed the clinical impacts of their variant to a loss of interaction (LOI) with proteins like FANCD2 and FOXN3. This mechanism could similarly apply to our variant (see **Figure 1C**), as it compromises the palm domain, affecting the Pocket region—a well-known site associated with interaction with most of the menin interaction proteins (MIPs) involved in the oncogenic process in MEN1 (12). However, since truncating menin proteins are frequently undetectable in MEN1 patients, frameshift variants like ours are likely subject to nonsense-mediated decay (NMD), positioning NMD as the primary disease mechanism in this context. Supporting this theory, our in silico analyses strongly predict NMD for the c.698dup variant, rather than LOI. This underscores the molecular diversity of frameshift variants in MEN1 and helps elucidate the variability in clinical presentations observed in families affected by MEN1.

The family described herein exhibits classical MEN1 phenotypes, including PHPT in 4 out of 5 carriers, and various neoplasms such as DP-NETs in 2 out of 5 carriers, and PitNENs in 2 out of 5 carriers (3). However, notable variations in phenotype were observed. Regarding DP-NETs, we observed an unusual deviation from the typical non-functional tumor presentation, which usually occurs at a rate of 40% (13). Notably, we identified two instances of functional NETs within a single family. Specifically, Case II-4 was diagnosed with Zollinger-Ellison syndrome at age 48.

Moreover, the index case initially presented with an insulinoma at 24, experienced a relapse at 25, and exhibited metastatic spread to two lymph nodes, with one testing positive for gastrin and ACTH. This case evolved into Cushing’s syndrome due to ectopic ACTH secretion syndrome (EAS) provoked by liver metastasis originating from a pancreatic neuroendocrine carcinoma (NEC), an occurrence not previously documented in MEN1 literature for pancreatic NECs. Ectopic ACTH secretion syndrome (EAS) is typically associated with bronchial, lung, and thymus neuroendocrine neoplasms (14) and is rare about DP-NETs. Thus, our index case underscores the significance of this clinical manifestation within the context of MEN1, presenting, to our knowledge, the first reported case of MEN1 with this specific clinical presentation.

Neuroendocrine carcinomas (NECs), whether small-cell (SCNECs) or large-cell (LCNECs), exhibit aggressive clinical behavior and poor prognosis, characterized by high-grade nuclear features, high mitotic counts (often >20 per 2 mm²), and a Ki-67 index usually exceeding 55% (15). They are rarely associated with hormonal syndromes. However, the index case features a functional ACTH-producing pancreatic-SCNEC (P-SCNEC), a rarity in MEN1-associated NETs, suggesting potential tumor grade progression from a well-differentiated DP-NET. This observation underscores the dynamic evolution of MEN1-associated tumors and highlights the complexity and heterogeneity of pNETs, including variations in histopathological grade, hormone secretion, and genetic alterations (13, 16). The phenomenon of grade progression, more common in metachronous than synchronous metastases, further emphasizes the evolving nature of these tumors (17). In our index case, this progression could be related to the rapid progression despite external radiotherapy and chemotherapy.

On the other hand, Pituitary Neuroendocrine Neoplasms (PitNENs) occur in 30% to 50% of individuals with MEN1 (18), typically emerging between the fourth and sixth decades (19). While PitNETs are usually macroadenomas (85%), our findings include two family members, III-3 and III-4, aged 23 and 25 respectively, with microadenomas, mainly as prolactinomas, and in one instance, with additional GH secretion, a rarity in only 5% of PitNETs (20). This finding of microadenomas, diverging from the typical macroadenoma presentation in MEN1, suggests the benefits of early detection through proactive screening. Early identification allows for more straightforward surgical management and minimizes neurological impacts, emphasizing the advantage of screening before the appearance of clinical symptoms for better management and outcomes.

This family report underscores the complexity and dynamic progression of MEN1, unveiling the identification of a functional ACTH-producing P-SCNEC as a novel entity within the spectrum of MEN1-associated DP-NETs. Additionally, we report the discovery of a pathogenic frameshift variant, c.698dup, in the MEN1 gene. This variant is subject to NMD, which may explain the varied clinical manifestations seen in this family.

While our findings emphasize the importance of cascade screening in facilitating early diagnosis and individualized treatment of MEN1, it is essential to note that our conclusions are based on a single-family cohort. Therefore, the significance of these findings should be considered with caution and in the context of broader studies. Nonetheless, proactive cascade genetic screening remains crucial for identifying asymptomatic carriers, optimizing patient care, and improving outcomes before the disease progresses to more critical stages.

## Data availability statement

The original contributions presented in the study are included in the article. Further inquiries can be directed to the corresponding author.

## Ethics statement

This study was approved by the Institutional Review Board of the Instituto Nacional de Cancerología. It was conducted in accordance with local legislation and institutional requirements. Written informed consent was obtained from the individuals for the publication of any potentially identifiable images or data included in this article.

## Author contributions

JR: Conceptualization, Data curation, Formal analysis, Methodology, Supervision, Validation, Visualization, Writing – original draft, Writing – review & editing. AG: Conceptualization, Formal analysis, Investigation, Supervision, Writing – original draft, Writing – review & editing. WT: Conceptualization, Data curation, Formal analysis, Supervision, Validation, Visualization, Writing – review & editing. VM: Methodology, Validation, Writing – review & editing. AR: Methodology, Validation, Writing – review & editing. IV: Data curation, Formal analysis, Investigation, Methodology, Writing – review & editing. JA: Data curation, Formal analysis, Investigation, Methodology, Writing – review & editing. JB: Data curation, Formal analysis, Investigation, Methodology, Writing – review & editing. JB: Data curation, Formal analysis, Investigation, Methodology, Writing – review & editing. OT: Data curation, Formal analysis, Investigation, Methodology, Writing – review & editing. LM: Data curation, Formal analysis, Investigation, Methodology, Supervision, Validation, Writing – review & editing.
